# Photoexcitation Dynamics of 4-Aminopthalimide in Solution Investigated Using Femtosecond Time-Resolved Infrared Spectroscopy

**DOI:** 10.3390/ijms252011038

**Published:** 2024-10-14

**Authors:** Hojeong Yoon, Seongchul Park, Raj Kumar Koninti, Manho Lim

**Affiliations:** Department of Chemistry and Chemistry Institute for Functional Materials, Pusan National University, Busan 46241, Republic of Korea; yun6793@naver.com (H.Y.); papagenona@gmail.com (S.P.); rajkumaryadav.k@gmail.com (R.K.K.)

**Keywords:** solution reaction dynamics, femtosecond time-resolved vibrational spectroscopy, solvent-assisted ESIPT, solvent reorganization, keto-to-enol tautomerization

## Abstract

Excited-state intramolecular proton transfer (ESIPT) reactions are crucial in photoresponsive materials and fluorescent markers. The fluorescent compound 4-aminophthalimide (4-AP) has been reported to exhibit solvent-assisted ESIPT in protic solvents, such as methanol, wherein the solvent interacts with 4-AP to form a six-membered hydrogen-bonded ring that is strengthened upon excitation. Although the controversial observation of ESIPT in 4-AP has been extensively studied, the molecular mechanism has yet to be fully explored. In this study, femtosecond infrared spectroscopy was used to investigate the dynamics of 4-AP in methanol and acetonitrile after excitation at 350 and 300 nm, which promoted 4-AP to the S_1_ and S_2_ states, respectively. The excited 4-AP in the S_1_ state relaxed to the ground state, while 4-AP in the S_2_ state relaxed via the S_1_ state without the occurrence of ESIPT. The enol form of 4-AP (Enol 4-AP) in the S_1_ state was calculated to be ~10 kcal/mol higher in energy than the keto form in the S_1_ state, indicating that keto-to-enol tautomerization was endergonic, ultimately resulting in no observable ESIPT for 4-AP in the S_1_ state. Upon the excitation of 4-AP to the S_2_ state, the transition to Enol-4-AP in the S_1_ state was found to be exergonic; however, ESIPT must compete with an internal conversion from the S_2_ to the S_1_ state. The internal S_2_ → S_1_ conversion was significantly faster than the solvent-assisted ESIPT, resulting in a negligible ESIPT for the 4-AP excited to the S_2_ state. The detailed excitation dynamics of 4-AP clearly reveal the molecular mechanism underlying its negligible ESIPT, despite the fact that it forms a favorable structure for solvent-assisted ESIPT.

## 1. Introduction

Excited-state intramolecular proton transfer (ESIPT) reactions have received growing attention due to their wide range of potential applications [1,2,3,4]. Typically, the ESIPT process is represented by a keto-to-enol tautomerization that occurs via a proton transfer reaction initiated by photoexcitation with short-laser pulses [5,6]. A detailed understanding of ESIPT is, therefore, crucial to achieve manipulation of the ESIPT dynamics for various applications [7,8,9], such as developing photo-responsive materials and bio-fluorescent markers.

**Scheme 1:**
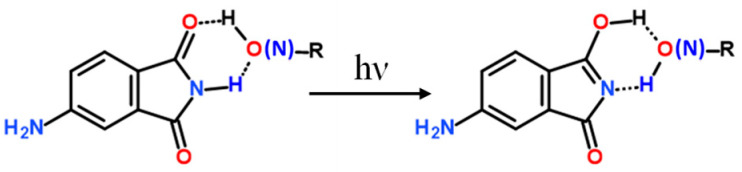
4-aminophthalimide (4-AP) can form a six-membered ring hydrogen bonding configuration with protic solvents.

The structure of 4-aminophthalimide (4-AP) contains electron acceptor moieties (>NH and –NH_2_) that are conjugated with an electron donor moiety (>C=O) in an aromatic ring. Consequently, its electron density changes significantly upon electronic excitation [10]. 4-AP can form a six-membered ring hydrogen bonding configuration with protic solvents, and this configuration can serve as a precursor for solvent-assisted ESIPT (Scheme 1). In addition, 4-AP is highly fluorescent in nonpolar solvents and its fluorescence quantum yield remains high (0.76–0.63) regardless of the polarity in aprotic solvents; however, the fluorescence yield drops dramatically in protic solvents [11]. The fluorescence spectra and lifetimes of 4-AP are also sensitive to the molecular environment [10,12,13,14,15,16]. Furthermore, 4-AP shares some important structural features with the nucleic acid bases, including the ability to undergo hydrogen bonding with protic solvent molecules and with itself. It has also been reported that 4-AP is approximately isosteric to tryptophan, a weakly fluorescent probe used in peptides and proteins, although the former exhibits a stronger fluorescence [17]. Consequently, 4-AP has been extensively employed as a strong fluorescent probe in various environments, including biological systems, nanomaterials, and polymers, since it can be readily incorporated into larger molecules without any significant changes in its fluorescence properties [10,16,18,19,20].

4-AP is known to exhibit strong solvatochromism. This was attributed to the significant increase in the dipole moment of its excited state relative to the ground state [21], the formation of stronger hydrogen bonds with protic solvents in the excited state [12,22], and/or the formation of the enol form of 4-AP (Enol-4-AP) as a result of solvent-assisted ESIPT [10,23,24,25]. ESIPT in 4-AP was revoked by showing the same behavior as 4-AP in 4-AP derivatives wherein an n-alkyl group replaces the imide hydrogen atom [26]. Later, Durantini et al. reported that 4-AP undergoes solvent-assisted ESIPT from the imino/amino group to the carbonyl group, leading to a keto–enol tautomerization in the excited state following excitation at 300 nm [10]. However, ESIPT of 4-AP in the excited state by excitation at 300 nm was also revoked by a comparative experiment on 4-AP and 4-AP derivatives wherein the hydrogen atom of the imino group is replaced by an *n*-butyl group [27]. Although the controversial observation of ESIPT in 4-AP has been extensively investigated, the molecular mechanism that defines the feasibility of this process has yet to be examined.

The electronic spectrum of 4-AP consists of two distinctive bands that lead to two different excited states (S_2_ and S_1_). While many studies have reported the solvatochromism behavior of 4-AP upon excitation to the S_1_ state, where the dipole moment and charge distribution change significantly upon excitation and, thus labeled ‘charge-transferred state’ [10,27], little efforts are made for the excitation to the S_2_ state where the dipole moment changes to a lesser extent (i.e., the non-charge transferred state). Previously, excitation to the S_2_ state has been suggested to result in keto–enol tautomerization, which was not observed upon excitation to the S_1_ state [10,27].

As expected, the choice of solvent plays a critical role in the excited-state dynamics of 4-AP. Protic solvents, such as methanol, can form hydrogen bonds with solute molecules, significantly influencing their electronic states and relaxation pathways. In addition, hydrogen bond interactions can stabilize specific excited states and facilitate processes such as ESIPT. In contrast, aprotic solvents, including acetonitrile, lack hydrogen bonding capabilities, providing a different interaction landscape for the solute. By comparing the behaviors of 4-AP in these two solvent environments, we can gain insights into the molecular mechanism of how solvent–solute interactions modulate the dynamics of the associated excited states.

Since molecular structural dynamics after photoexcitation are quite crucial for understanding the excited state properties, direct experimental observation of the structural dynamics is necessary to unveil the mechanistic details of environment-induced excited state properties. In this context, time-resolved infrared (TRIR) spectroscopy can detect structural dynamics in their excited states, in addition to proton coordination during the ESIPT processes. This is possible because TRIR spectroscopy is sensitive not only to the molecular structure but also to the solvation environment [28,29]. Femtosecond TRIR spectroscopy has been utilized in obtaining a molecular picture of the photophysical and photochemical pathways involved in the excitation of interested compounds in solution [30,31,32,33,34].

In this study, the dynamics of photoexcited 4-AP in methanol and acetonitrile are investigated using femtosecond TRIR spectroscopy to explore the mechanistic feasibility of ESIPT in 4-AP. Excitation wavelengths of 300 and 350 nm are used to excite the 4-AP molecule to the S_2_ and S_1_ states, respectively, and the resulting structures are probed with mid-IR pulses in the spectral region of 1800–1450 cm^−1^, where the spectral signatures of the related compounds are distinctive. Deuterated solvents (i.e., CD_3_OD and CD_3_CN) are subsequently used to shift the solvent absorption away from the spectral window of interest. Moreover, a solution 4-AP in CH_3_OH is also studied in a limited spectral region to test the isotope effect in the presence of ESIPT; this limited spectral region is required due to overlap with the strong solvent absorption. Detailed excitation dynamics are obtained to explain the negligible ESIPT of 4-AP in protic solvents.

## 2. Results and Discussion

**Figure 1:**
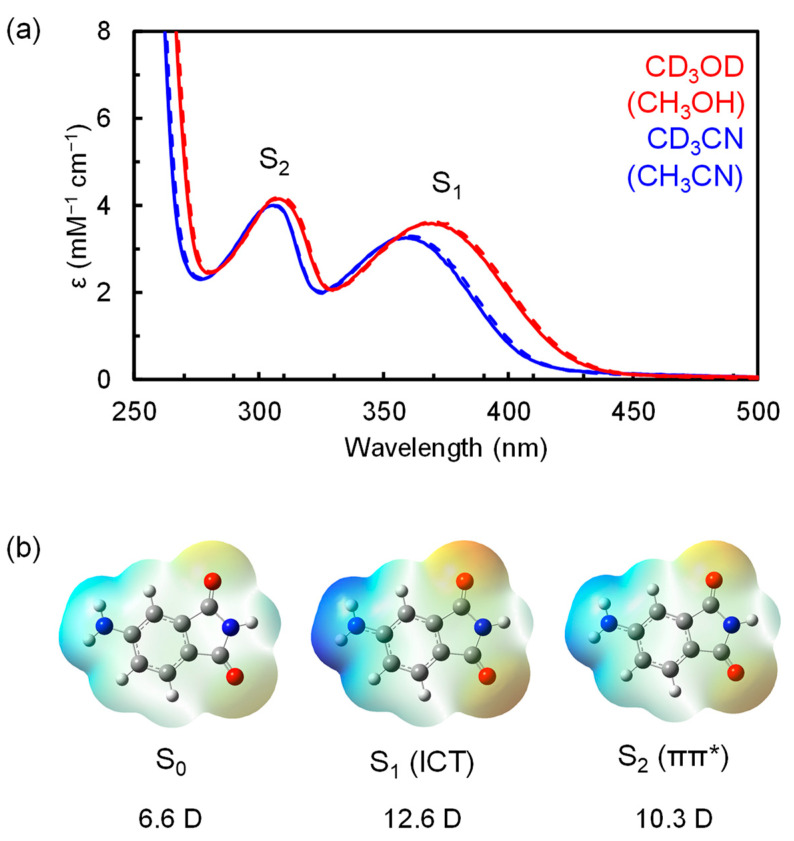
(**a**) Electronic absorption spectra of 4-AP in CD_3_OD (solid red line), CH_3_OH (dashed red line), CD_3_CN (solid blue line), and CH_3_CN (dashed blue line) at room temperature. Two bands were assigned to the transitions to the S_1_ and S_2_ states, as shown in the figure. (**b**) ESPs of the S_0_, S_1_, and S_2_ states obtained from TD-DFT calculations using the ωB97XD functional and the pcseg-1 basis set. The blue and red clouds represent more positive and negative potentials, respectively. The S_1_ state is more polarized and significantly different from the S_0_ state, consistent with the assignment of the S_1_ state to the ICT state. Dipole moments calculated using the ωB97XD/pcseg-1 are also given.

The UV-Vis absorption spectra of 4-AP in both undeuterated and deuterated methanol and acetonitrile are shown in Figure 1a. It can be seen that the use of a deuterated solvent did not alter the UV-Vis spectra, implying that the electronic transitions of 4-AP are not affected by deuteration. In both solvents, the absorption spectra consist of two prominent bands that correspond to two different transitions, namely the band at ~310 nm, which is assigned to the S_0_ → S_2_ (ππ*) transition, and the band at ~360 nm, which is attributed to the S_0_ → S_1_ intramolecular charge transfer (ICT) transition [10]. Although the position of the ~310 nm band is similar in both solvents (~2 nm difference), the position of the band at ~360 nm changes significantly, exhibiting a 12 nm difference between acetonitrile and methanol. This observation is consistent with previous reports, wherein band positions of 306.2 and 357.1 nm were reported for acetonitrile, and positions of 308.2 and 369.2 nm were reported in methanol [10]. For 4-AP, the transition occurring at ~360 nm is known to result in a significant change in the dipole moment, which is manifested as a large solvatochromism [10] Using time-dependent density functional theory (TD-DFT) calculations with the ωB97XD functional and the pcseg-1 basis set, the dipole moment and electrostatic potential (ESP) of 4-AP were determined in the S_0_, S_1_, and S_2_ states. According to these calculations, the dipole moment of 4-AP changes by 6.0 D for the S_1_ state and by 3.7 D for the S_2_ state upon excitation. The ESP of 4-AP shown in Figure 1b displays that the charge distribution in the S_1_ state is considerably more polarized compared to the S_0_ and S_2_ states. Although methanol and acetonitrile have similar polarity with dielectric constants of 32.63 and 36.69, respectively, at 298K [35,36], only the protic solvent methanol can form a hydrogen bond with 4-AP. Therefore, the larger redshift of the S_1_ peak of 4-AP in methanol was attributed to a greater stabilization of the more polarized S_1_ state by the hydrogen bonding of 4-AP with methanol. The S_1_ peak is significantly more sensitive to the hydrogen bonding ability of the solvent than the S_2_ peak [10].

**Figure 2:**
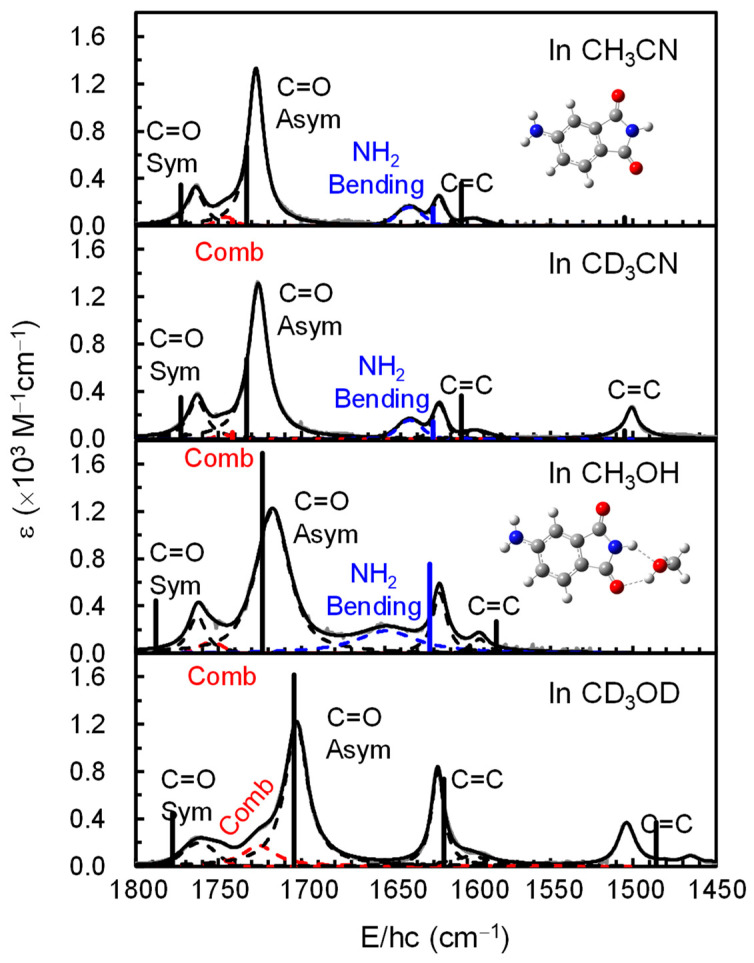
Equilibrium vibrational spectra of 4-AP (data and fit shown in grey and black sold lines, respectively) in undeuterated and deuterated acetonitrile and methanol at room temperature. The absorption spectra were assigned to the symmetric (Sym) and asymmetric (Asym) C=O stretching modes and the C=C stretching modes of the benzene ring (black dashed lines). The NH_2_ bending mode (blue dashed lines) is not present for the solution of 4-AP in CD_3_OD, wherein the amine moiety is deuterated and the ND_2_ bending mode is outside of the spectral window shown. The calculated vibrational frequencies are shown as vertical lines with scale factors of 0.923–0.975. Additionally, combination bands (Comb) of NH_2_ scissoring (or C–N–C stretching in CD_3_OD) and OC–N–CO scissoring (red dashed lines), were assigned. The asymmetric C=O stretching mode exhibits a red shift in the presence of hydrogen bonding between 4-AP and methanol. The calculated optimized molecular structures are shown for 4-AP with and without hydrogen bonding to CH_3_OH.

Figure 2 shows the equilibrium Fourier transform infrared (FT-IR) spectra of 4-AP in undeuterated and deuterated acetonitrile and methanol at room temperature. It was found that CH_3_CN and CH_3_OH exhibit strong absorption bands at ~1400 cm^−1^ due to the CH_3_ bending mode, which also produces an absorbance of >0.5, even at 1550 cm^−1^ under the current conditions. As a result, the measured sample absorbance is unreliable below 1550 cm^−1^ and so the data for this spectral region are not shown for 4-AP in CH_3_CN and CH_3_OH. Consequently, deuterated solvents were used to extend the spectral region up to 1450 cm^−1^. Although the deuterated solvent altered the vibrational spectrum of 4-AP in methanol, the vibrational spectrum of 4-AP in CD_3_CN was almost identical to that in CH_3_CN. Based on these results, it is apparent that the electronic and vibrational transitions of 4-AP are not affected by the deuteration of acetonitrile; thus, only the solution of 4-AP in CD_3_CN was considered hereafter for the sake of simplicity. In contrast, solutions of 4-AP in both CD_3_OD and CH_3_OH are investigated due to the differences in their vibrational spectra. The two major bands at 1763 and 1703 cm^−1^ in CD_3_OD, 1763 and 1718 cm^−1^ in CH_3_OH, and 1763 and 1726 cm^−1^ in CD_3_CN correspond to the symmetric and asymmetric C=O stretching modes of 4-AP, respectively. The band positions for the symmetric C=O stretching mode are identical in acetonitrile and methanol, whereas the bands corresponding to the asymmetric C=O stretching mode were found to be solvent dependent. DFT calculations performed for 4-AP in the presence of one methanol molecule showed that methanol is hydrogen bonded to 4-AP (optimized structure in Figure 2). In addition, these calculations show that the asymmetric C=O stretching mode undergoes a red-shift, indicating that this band originates from hydrogen bonding between methanol and the C=O and imine moieties of 4-AP. The asymmetric C=O band of 4-AP in CD_3_OD is even more red-shifted than that in CH_3_OH due to the deuteration of the 4-AP amine and imine groups. As can be seen in Figure 2, the signals corresponding to NH_2_ bending of 4-AP in CD_3_CN and CH_3_OH (i.e., at 1635 and 1649 cm^−1^, respectively) are absent in CD_3_OD due to the deuteration of the amine group. The remaining bands at 1618, 1598, and 1504 cm^−1^ in CD_3_OD, 1617 and1593 cm^−1^ in CH_3_OH, and 1617, 1597, and 1501 cm^−1^ in CD_3_CN correspond to the C=C stretching modes of the 4-AP benzene ring. The small band at ~1750 cm^−1^ (red dashed lines in Figure 2) was assigned to a combination of NH_2_ scissoring (or C–N–C stretching in CD_3_OD) and OC–N–CO scissoring. These distinctive bands of 4-AP in the 1800–1450 cm^−1^ region were used as reference peaks in the vibrational analysis of the excited state due to the fact that they are observable in the same region across all IR spectra, even in the presence of the tautomerized compound.

**Figure 3:**
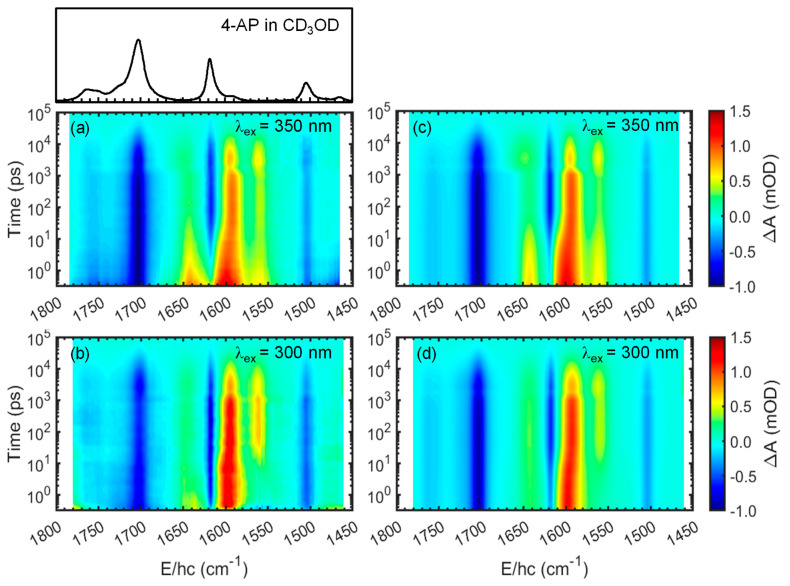
2D Contour maps of the TRIR spectra of a 10 mM 4-AP solution in CD_3_OD at 293 K after excitation at (**a**) 350 and (**b**) 300 nm. The corresponding simulated TRIR spectra at (**c**) 350 and (**d**) 300 nm are also shown. The TRIR spectra at 350 nm were fitted using the three basis spectra of the S_0_, S_1-0_, and S_1-1_ states, while the TRIR spectra at 300 nm were fitted using the three basis spectra of the S_0_, S_1-1_, and S_2_ states shown in Figure 5a (see text). The pump-induced absorbance change, ΔA(*ῡ*, *t*), was obtained as a function of the probe wavenumber (*ῡ*) and the pump-probe delay time (*t*) by subtracting the absorbance of the sample before excitation from that after excitation. ΔA(*ῡ*, *t*) is expressed in the units of mOD, wherein 1 mOD = 10^−3^ OD (optical density). The equilibrium FT-IR spectrum is also shown above panel (**a**).

**Figure 4:**
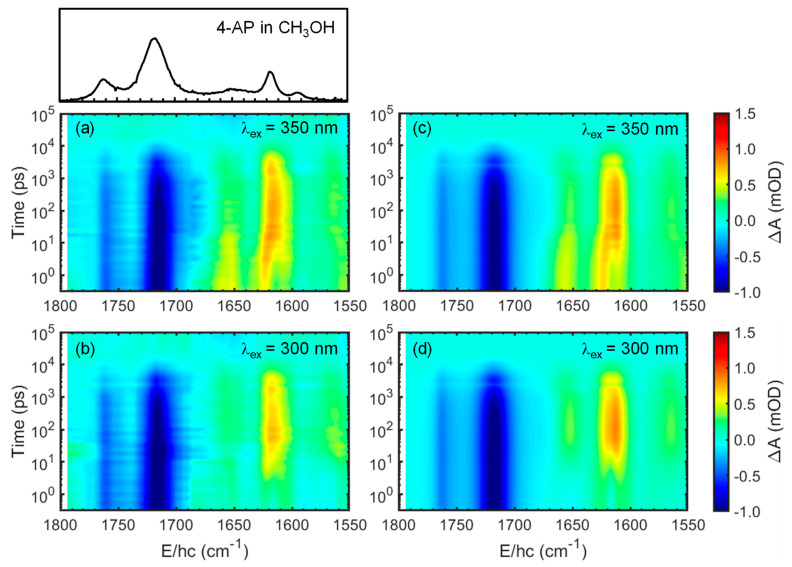
2D Contour maps of the TRIR spectra of a 10 mM 4-AP solution in CD_3_OH at 293 K after excitation at (**a**) 350 and (**b**) 300 nm. The corresponding simulated TRIR spectra at (**c**) 350 and (**d**) 300 nm are also shown. The TRIR spectra at 350 nm were fitted using the three basis spectra of the S_0_, S_1-0_, and S_1-1_ states, while the TRIR spectra at 300 nm were fitted using the three basis spectra of the S_0_, S_1-1_, and S_2_ states shown in Figure 5a (see text). Note that the spectral range is narrowed to 1800–1550 cm^−1^ compared with the CD_3_OD system due to a strong solvent absorption of CH_3_OH in the spectral region < 1550 cm^−1^. The pump-induced absorbance change, ΔA(*ῡ*, *t*), was obtained as a function of the probe wavenumber (*ῡ*) and the pump-probe delay time (*t*) by subtracting the absorbance of the sample before excitation from that after excitation. ΔA(*ῡ*, *t*) is expressed in the units of mOD, wherein 1 mOD = 10^−3^ OD (optical density). The equilibrium FT-IR spectrum is also shown above panel (**a**).

One key objective of this work was to establish a detailed picture of the roles of different electronic states in the solvent-assisted ESIPT process of 4-AP in protic solvents. The electronic absorption spectra (Figure 1) demonstrated the possibility of selective photoexcitation from the S_0_ state of 4-AP to either the S_2_ or S_1_ state. The TRIR spectra of 10 mM 4-AP solutions in CD_3_OD and CH_3_OH were recorded at 293 K over the spectral ranges of 1800–1450 and 1800–1550 cm^−1^, respectively. More specifically, the spectra were recorded from 0.3 ps to 100 ns after excitation with a 350 or 300 nm pulse, and the resulting two-dimensional (2D)-contour maps are displayed in Figure 3 and Figure 4. The TRIR spectra clearly display evolving negative-going (blue) and positive-going (red) features. The negative-going features (bleach), appearing immediately after photoexcitation and having the same band positions as the equilibrium absorption spectrum of 4-AP, arise from population depletion of the ground state upon photoexcitation. The amplitudes of the bleach bands decreased over time, essentially disappearing within 100 ns, suggesting that the depleted ground-state population recovered within this timeframe. Some new absorption features also developed immediately after photoexcitation, while others appeared later. However, all absorption features were observed to decay during the bleach recovery time, suggesting that all excited states relax or new species return to the reactant within the 100 ns period.

**Figure 5:**
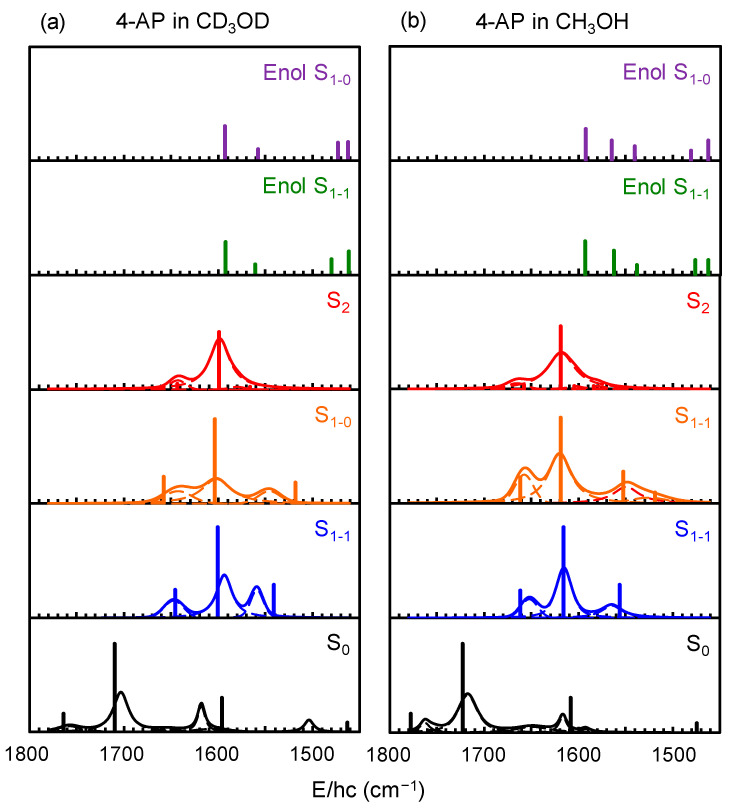
Basis spectra used to fit the TRIR spectra of 4-AP in (**a**) CD_3_OD and (**b**) CH_3_OH excited at 350 and 300 nm. Basis spectrum S_0_ is the equilibrium FT-IR spectrum of 4-AP in CD_3_OD or CH_3_OH. The S_2_, S_1-0_, and S_1-1_ basis spectra were obtained by optimizing the calculated vibrational band (vertical lines with scale factors of 0.923–0.975) to globally fit the TRIR spectra of 4-AP in CD_3_OD or CH_3_OH. The basis spectra S_1-0_ and S_1-1_ were calculated for 4-AP in methanol before and after, respectively, optimization of the solvent configuration upon excitation to the S_1_ state. The calculated Enol S_1-0_ and Enol S_1-1_ basis spectra are also shown with the same scale factor as S_1-0_ for comparison.

The TRIR spectra were globally fitted using the basis spectra shown in Figure 5 by adjusting the amplitude of the basis spectra whilst maintaining the center frequency and width of each band in the spectra constant. The basis spectra were assigned to specific chemical species based on quantum calculations and possible reaction dynamics. In addition to the equilibrium spectrum of 4-AP (denoted as S_0_), the use of two additional basis spectra that were assigned to 4-AP in the S_1_ state was sufficient to reproduce the TRIR spectra at 350 nm. More specifically, these spectra included one basis spectrum (denoted as S_1-0_) for 4-AP in the S_1_ state immediately after photoexcitation and before the solvent reorganizes to a new electronic configuration of the S_1_ state, and a second basis spectrum (denoted as S_1-1_) for 4-AP in the S_1_ state with the fully optimized solvent configuration for the S_1_ state. The TRIR spectra at 300 nm, which were excited to the S_2_ state, also required three basis spectra, namely S_0_, S_1-1_, and S_2_ shown in Figure 5. The two basis spectra S_0_ and S_1-1_ for the TRIR spectra at 300 nm were the same as those used to fit the TRIR spectra at 350 nm. Although the S_1-0_ and S_1-1_ basis spectra were required for the excitation of 4-AP to the S_1_ state, one basis spectrum was sufficient for the excitation of 4-AP to the S_2_ state. This can be attributed to the fact that the spectrum for the S_2_ state immediately after photoexcitation is approximately the same as that for 4-AP in the S_2_ state with the fully adjusted solvent configuration. As can be seen in Figure 1b, the ESP of 4-AP in the S_2_ state is not significantly different from that in the S_0_ state, and thus, the solvent configuration does not change significantly after the transition of S_0_ → S_2_, resulting in little difference between the spectra in the S_2_ state before and after optimization of the solvent configuration. In the case of excitation to the S_1_ state, the ESP changes dramatically; thus, the spectra before and after optimization (i.e., S_1-0_ and S_1-1_) are required. In the global fitting of both TRIR spectra at 350 and 300 nm in a given solvent, two basis spectra, S_0_ and S_1-1_ were common. As shown in Figure 3 and Figure 4, the TRIR spectra at 350 and 300 nm in CD_3_OD and CH_3_OH were well reproduced by the sum of the three basis spectra (i.e., S_0_, S_1-0_, and S_1-1_ for 350 nm; S_0_, S_1-1_, and S_2_ for 300 nm) of the corresponding solvent in Figure 5.

The vibrational frequencies of 4-AP in various electronic states were calculated using the DFT method with the ωB97XD functional and the pcseg-1 basis set. The basis spectrum of S_0_ represents the equilibrium FT-IR spectrum of 4-AP in methanol. The S_2_, S_1-0_, and S_1-1_ basis spectra were obtained by optimizing the calculated vibrational frequencies of 4-AP in the corresponding states. The calculated vibrational spectra consisted of major bands corresponding to the C=O and C=C stretching modes. The position and width of each band were optimized to globally fit the TRIR spectra. The basis spectra of S_1-0_ and S_2_ were optimized during global fitting of the TRIR spectra at 350 and 300 nm, respectively, and the basis spectrum of S_1-1_ was optimized during simultaneous global fitting of the two TRIR spectra at 350 and 300 nm. Initially, the integrated area of each band in the basis spectra was kept proportional to the calculated oscillator strength in the global fitting and was then slightly optimized in the final fitting.

The spectrum for S_1-0_ was calculated using the solvent configuration in the S_0_ state, whilst the 4-AP molecule was in the S_1_ state. The spectrum for S_1-1_ was calculated after the solvent configuration was fully optimized for 4-AP in the S_1_ state using TD-DFT. The same notation as that for the S_1_ state can be used for 4-AP in the S_2_ state, i.e., S_2-0_ and S_2-2_ were calculated immediately after excitation from the S_0_ state to the S_2_ state, and with the fully optimized solvent configuration in the S_2_ state, respectively. The spectrum of S_2-2_ was indistinguishable from that of S_2-0_; thus, the spectra were denoted as S_2_ in both cases. The Gibbs free energy of 4-AP for the S_2-2_ state was calculated to be 0.11 kcal/mol lower than the S_2-0_ state, resulting in the S_2-2_ spectrum being insignificantly different from that of the S_2-0_ state. However, the Gibbs free energy of 4-AP for the S_1-1_ state was calculated to be 1.8 kcal/mol lower than that of the S_1-0_ state, resulting in the S_1-1_ spectrum being significantly different from that of the S_1-0_ state. The insignificant difference between the spectra of S_2-0_ and S_2-2_ allowed a single S_2_ spectrum to be used in both cases.

To explore the possible formation of Enol 4-AP after photoexcitation via solvent-assisted ESIPT, the spectra of Enol 4-AP were calculated in the S_1_ state before and after full optimization of the solvent configuration to Enol 4-AP in the S_1_ state, denoted as Enol S_1-0_ and Enol S_1-1_, respectively. The basis spectra denoted as S_0_, S_1-0_, S_1-1_, and S_2_ represent the spectra of the keto form of 4-AP in various electronic states. As shown in Figure 5, the calculated spectral features of Enol 4-AP in the S_1_ state are quite different from those of the keto form, being significantly red-shifted and showing congestion in the lower frequency region. These observations indicate that, when present, Enol 4-AP can be readily distinguishable in the TRIR spectra. No distinct absorption features were observed in the TRIR spectral region < 1540 cm^−1^ (see Figure 3) wherein the presence of Enol 4-AP should be evident. This indicates that ESIPT does not proceed in a methanolic solution of 4-AP excited at either 350 or 300 nm.

Global fitting of the TRIR spectra using the basis spectra resulted in time-dependent amplitude changes in the basis spectra, which reflected population changes in the corresponding species after the photoexcitation of 4-AP. The amplitude of the basis spectrum represents the sum of the integrated areas of the bands in the basis spectra used to fit the TRIR spectra and is proportional to the sum of the integrated extinction coefficients of the bands in the spectrum multiplied by the population of the corresponding species. Thus, the time-dependent population changes in the species can be obtained from the recovered time-dependent amplitude changes in the basis spectra used to fit the TRIR spectra once the integrated extinction coefficients of the corresponding basis spectra are determined [37].

**Figure 6:**
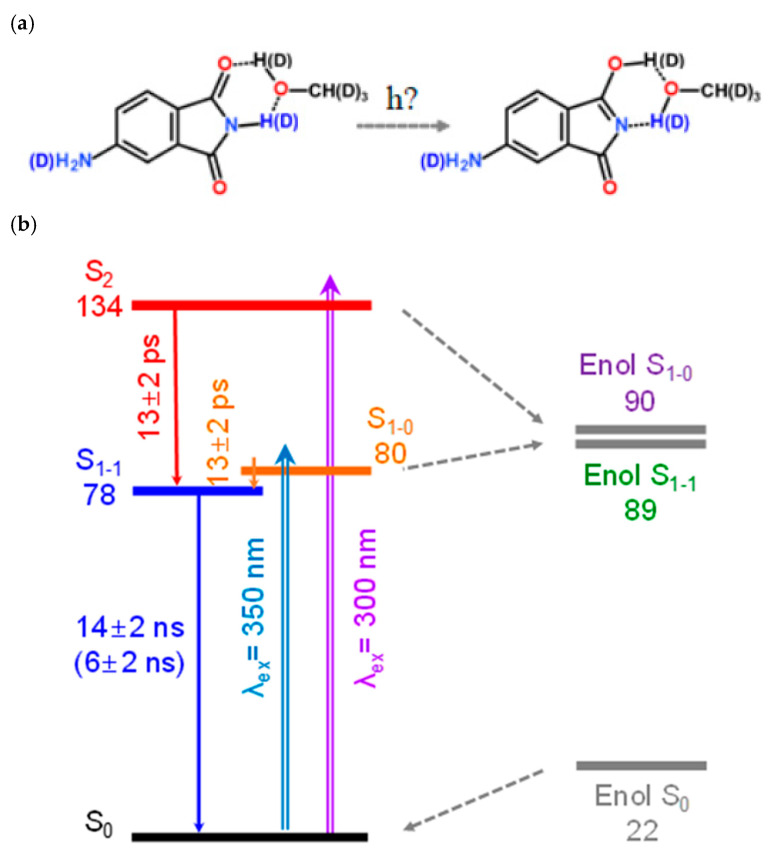
(**a**) Structural formulae of the keto and enol forms 4-AP, each hydrogen bonded to a methanol molecule (CH_3_OH or CD_3_OD). Amines of 4-AP are also deuterated in CD_3_OD. Enol 4-AP is generated by the solvent-assisted ESIPT. (**b**) Kinetic model that simultaneously describes the reaction dynamics of 4-AP in methanol excited at 350 and 300 nm. In the ground state, 4-AP is present in the keto form (S_0_), and the tautomerization of the keto form can generate the enol form. The possible tautomerization pathways are represented by gray dashed arrows. The time constant for each process was obtained by simultaneously fitting both time-dependent amplitude changes in the basis spectra from the global fittings of the TRIR spectra of 4-AP in methanol excited at 350 and 300 nm (Figure 7). All time constants were comparable in both CD_3_OD and CH_3_OH, with the exception of the relaxation time of the S_1-1_ → S_0_ transition, wherein the value in CH_3_OH is given in parentheses. The calculated relative Gibbs free energy of each state referenced to the S_0_ state is given in kcal/mol. Although the calculated energy of the S_2_ state is higher than the excitation energy at 300 nm (95 kcal/mol), the electronic spectrum of 4-AP clearly shows that the 300-nm excitation results in the S_2_ state. The absolute value of the calculated energy for the excited electronic state may have a large error. The quoted uncertainties are the experimental and fitting errors.

The kinetic model (Figure 6b) was used to describe the reaction dynamics of excited 4-AP in methanol. This model was selected due to its ability to reproduce the time-dependent amplitude changes in the basis spectra obtained from the global fitting of the TRIR spectra at both 350 and 300 nm. The pump wavelength-dependent and time-dependent fractional population changes were obtained by simultaneously fitting the time-dependent amplitude changes for both the 350 and 300 nm excitations to the kinetic model by adjusting the integrated extinction coefficients of all species and rate constants for all processes between species. As shown in the figure, the time-dependent fractional population changes in all the species involved in the photoexcitation of 4-AP at 350 and 300 nm, as obtained from the corresponding time-dependent amplitude changes in the basis spectra and the fitted integrated extinction coefficients of the corresponding species, were well reproduced by the kinetic model with the time constants shown in the Figure 6b. It can be seen from Figure 6b that the S_1-1_ → S_0_ relaxation is common in a given solvent for excitation at both 350 and 300 nm. In other words, the time constants of the S_1-1_ → S_0_ relaxation are the same for 4-AP in a given solvent excited at both 350 and 300 nm. Time constants for the S_2_ → S_1-1_ and the S_1-0_ → S_1-1_ relaxations are identical, which happens to be the same value of 13 ± 2 ps, for 4-AP in both CD_3_OD and CH_3_OH, while the S_1-1_ → S_0_ relaxation was found to be ~2.3-times faster in CH_3_OH than CD_3_OD. The fitted relative integrated extinction coefficients obtained from Figure 6b were 0.45 ± 0.02, 0.95 ± 0.02, and 0.94 ± 0.02 for the S_2_, S_1-1_, and S_1-0_ spectra relative to that of S_0_, respectively. Notably, these correlate with the calculated values of 0.45, 0.95, and 0.94 for the S_2_, S_1-1_, and S_1-0_ states, respectively.

As can be seen in Figure 6b, the solvent configuration is fully optimized to 4-AP in the S_1_ state with a time constant of 13 ± 2 ps. This state remains until it relaxes into the S_0_ state with time constants of 14 ± 2 and 6 ± 2 ns in CD_3_OD and CH_3_OH, respectively. Since all excited 4-AP species are in the S_1_ state upon 350 nm excitation, the excited 4-AP at this wavelength undergoes a simple relaxation to the S_0_ state following picosecond optimization of the solvent configuration to 4-AP in the excited S_1_ state (Figure 6b). The excitation of 4-AP at 350 nm represents an ICT transition, which results in a significant change in the dipole moment (∆μ ≈ 6 D) and the ESP (Figure 1). Immediately after photoexcitation, 4-AP reaches the S_1_ state, residing in non-relaxed solvent environments since the solvent molecule configuration remains in the S_0_ state. As the solvent molecules become reorganized based on the new charge distribution of 4-AP in the S_1_ state, 4-AP becomes stabilized. The time constant of 13 ± 2 ps for the S_1-0_ → S_1-1_ transition, therefore, represents the solvent reorganization time of methanol and is comparable to the dielectric relaxation time of methanol measured by the time-resolved fluorescence Stokes shift method (i.e., 17 ps) [38], thereby consolidating the current experimental data. The solvent reorganization time of CD_3_OD was identical to that of CH_3_OH, as was its internal conversion time from the S_2_ to the S_1_ state of 4-AP. As shown in Figure 5, solvent reorganization manifested as spectral evolution and dynamic peak shifts of the bands in the S_1-0_ to S_1-1_ basis spectra. Fluorescence decay measurements identified the excited state lifetime of 4-AP in CH_3_OH to be ~7 ns [11], which was proposed to be ~3 times lower than that in CD_3_OD [27]. Therefore, the S_1-1_ lifetimes in CD_3_OD and CH_3_OH (i.e., 14 ± 2 and 6 ± 2 ns, respectively) were consistent with the previously reported values.

The fluorescence quantum yield of 4-AP in CH_3_OH was reported to be 0.1 [39], while that in CD_3_OD has been reported as ~0.3 [27]. The longer lifetime of the S_1_ state of 4-AP in CD_3_OD supports a higher fluorescence quantum yield, indicating that the shorter lifetime of the S_1_ state in CH_3_OH arises from faster nonradiative relaxation. The solvent deuterium effect on the fluorescence lifetime was attributed to the participation of the solvent proton in the nonradiative decay channels of the excited molecule [27].

According to Figure 6b, the 4-AP S_2_ state generated upon 300 nm excitation relaxes into the S_1_ state via internal conversion with a time constant of 13 ± 2 ps. Optimization of the solvent configuration to the S_2_ state was undetectable because the spectrum of the S_2_ state with the optimized solvent configuration was indistinguishable from that with the pre-optimized solvent configuration. Because the internal conversion time from the S_2_ to the S_1_ state is comparable to, happens to be the same as, the solvent reorganization time of the S_1_ state, the spectrum for the S_1_ state with a pre-optimized solvent configuration (S_1-0_) is not observable during internal conversion from the S_2_ to the S_1_ state. Therefore, the simple relaxation scheme of S_2_ → S_1-1_ → S_0_ is sufficient for describing the decay of 4-AP excited to the S_2_ state.

It has been reported that substituting hydrogen for deuterium preserves the electrostatic potential but significantly slows the ESIPT process [40]. In the current study, the relaxation times of the initially excited states at 300 or 350 nm were identical in both CD_3_OD and CH_3_OH. If proton transfer was involved in these relaxation processes, the time constant would depend on solvent deuteration. However, the independence of the initial relaxation time on solvent deuteration confirmed that proton transfer was not involved in the initial evolution of the TRIR spectra at 300 and 350 nm.

Considering the structure presented in Figure 6a, wherein 4-AP is hydrogen bonded to a methanol molecule in the ground state, with CH_3_OH bridging between the C=O and imide N–H moieties of 4-AP, the excited 4-AP possesses an optimal precursor configuration for solvent-assisted ESIPT. Furthermore, the charge of the accepting oxygen increases (see the ESPs in Figure 1), thereby strengthening the hydrogen bonds in the 4-AP–methanol complex and enhancing the probability of proton transfer upon excitation to the S_1_ state [24,41]. Therefore, the S_1_ state of 4-AP is favored in the context of solvent-assisted ESIPT. However, as shown in Figure 6, the calculated Gibbs free energy of the Enol S_1_ state is ~10 kcal/mol higher than that of the keto-form S_1_ state. Consequently, the lack of solvent-assisted ESIPT in the S_1_ state of 4-AP arises for energetic reasons. The majority of ESIPT processes have been observed for enol-to-keto tautomerizations due to the fact that the excited enol is higher in energy [4,42]. This indicates that the ESIPT of 4-AP requires an uncommon keto-to-enol tautomerization to proceed [40]. The higher energy of Enol 4-AP in the S_1_ state is consistent with the majority of molecules possessing higher energy in this form and state. Upon the excitation of 4-AP to the S_2_ state, the energy of which is higher than that of the Enol S_1_ state, the energetics of ESIPT are satisfied. However, the proton transfer process from the S_2_ state should compete with the S_2_ → S_1_ internal conversion with a time constant of 13 ± 2 ps. In other words, proton transfer should proceed with a comparable time of 13 ps to be successful, requiring the acidity and basicity of the related atoms to increase and the hydrogen bonding configuration with the solvent molecule to be optimized. However, the ESP of the S_2_ state is similar to that of the S_1_ state, indicating that hydrogen bonding is not dramatically strengthened in the S_2_ state compared to the S_0_ state. Therefore, proton transfer is slower than the internal conversion time of 13 ps, resulting in a negligible ESIPT for 4-AP in the S_2_ state.

**Figure 8:**
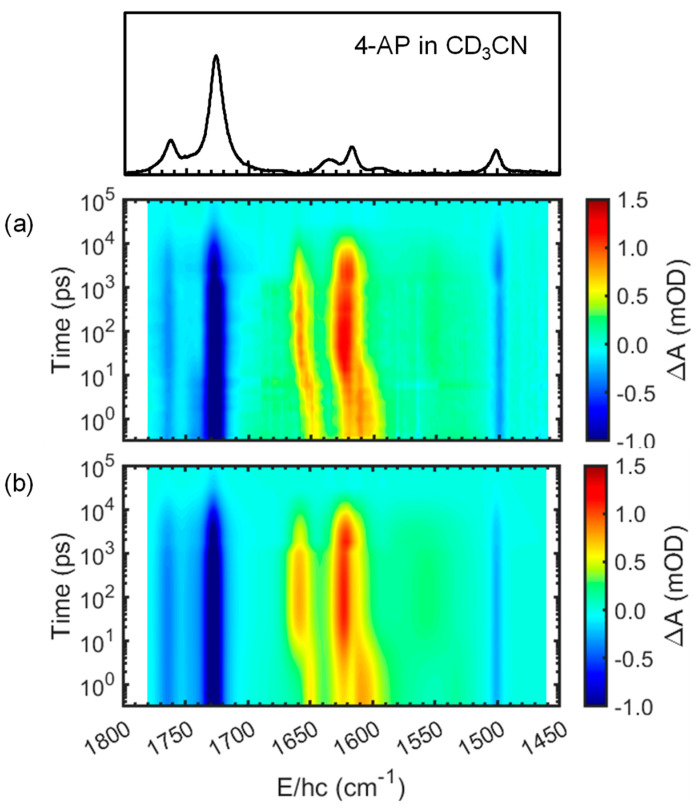
(**a**) 2D Contour map for the TRIR spectra of a 10 mM solution of 4-AP in CD_3_CN at 293 K after excitation at 300 nm. (**b**) Simulated TRIR spectra obtained using the basis spectra shown in Figure 9a (see text). The pump-induced absorbance change, ΔA(*ῡ*, *t*), was obtained as a function of the probe wavenumber (*ῡ*) and the pump-probe delay time (*t*) by subtracting the absorbance of the sample before excitation from that after excitation. ΔA(*ῡ*, *t*) is expressed in the units of mOD, wherein 1 mOD = 10^−3^ OD (optical density). The equilibrium FT-IR spectrum is also shown above panel (**a**).

**Figure 9:**
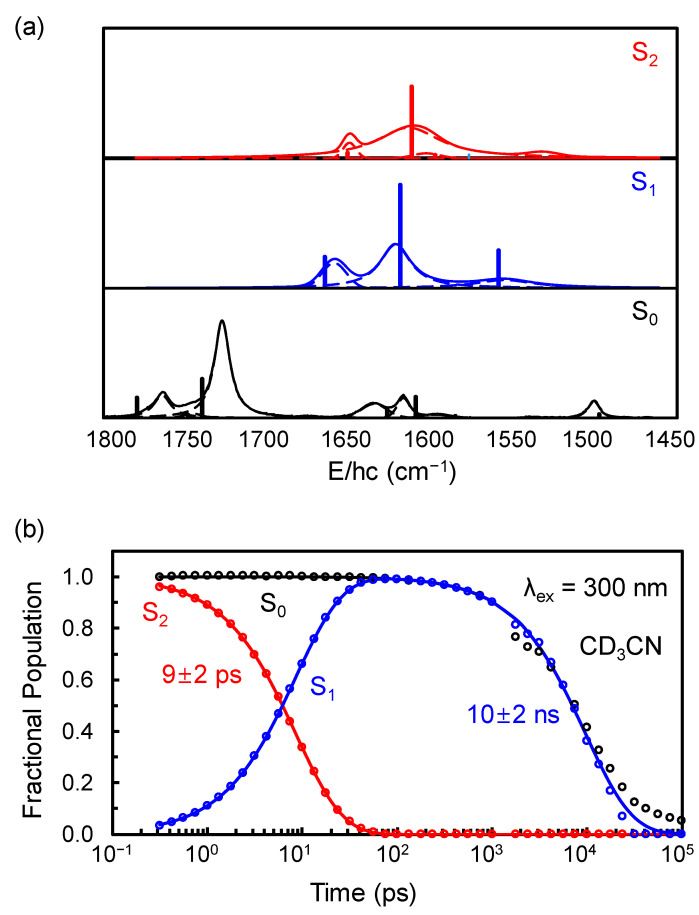
(**a**) Basis spectra used to fit the TRIR spectra of 4-AP in CD_3_CN excited at 300 nm. The basis spectrum for S_0_ represents the equilibrium FT-IR spectrum of 4-AP in CD_3_CN. The basis spectra for the S_2_ and S_1_ states were obtained by optimizing the calculated vibrational band (vertical lines with scale factors of 0.937–0.975) in acetonitrile to globally fit the TRIR spectra of 4-AP in CD_3_CN. The spectra denoted as S_1_ and S_2_ represent the spectra of 4-AP with a fully optimized solvent configuration to its corresponding electronic state since the solvent reorganization time of acetonitrile is ~0.2 ps [43]. (**b**) Time-dependent fractional population changes in the various species involved in the excitation of 4-AP in CD_3_CN at 300 nm. The fractional population changes are color-coded: The red circles and line represent the fractional population of 4-AP in CD_3_CN in the S_2_ state, while the blue elements represent the fractional population in the S_1_ state. The black circles and line represent the fractional population change in the depleted 4-AP in CD_3_CN after excitation at 300 nm. Data for the fractional population changes (open circles), were obtained from the time-dependent amplitude changes in the basis spectra and the fitted integrated extinction coefficients of the corresponding basis spectra. These data are well described by the Figure 6b shown in Figure 6b with time constants of 9 ± 2 ps and 10 ± 2 ns for the S_2_ → S_1_ and S_1_ → S_0_ transitions, respectively (solid lines).

To differentiate between the possible solvent-assisted ESIPT of 4-AP in the excited S_2_ state, the dynamics of 4-AP were investigated in a polar aprotic solvent excited at 300 nm. More specifically, the TRIR spectra of a 10 mM 4-AP solution in CD_3_CN were obtained between 1800 and1450 cm^−1^ and at 293 K over a wide time range of 0.3 ps to 100 ns after excitation with a 300 nm pulse; the corresponding 2D contour map is shown in Figure 8. As described above, the early dynamics of 4-AP are expected to be the same in CD_3_CN and CH_3_CN because the electronic and vibrational spectra of 4-AP are not affected by deuteration. Since the spectral window is broader in CD_3_CN, experiments were performed for a solution of 4-AP in this solvent. From Figure 8, it can be seen that the negative-going features, which appeared immediately after photoexcitation, matched well with the bands in the equilibrium FT-IR spectrum of 4-AP in CD_3_CN; positive-going features, appearing immediately or later, evolved over time. The TRIR spectra were globally fitted using the three basis spectra presented in Figure 9a, namely the equilibrium FT-IR spectrum of 4-AP in CD_3_CN (S_0_), the spectrum of 4-AP in the S_1_ state (S_1_), and the spectrum of 4-AP in the S_2_ state (S_2_). Since the dielectric relaxation time of acetonitrile is known to be ~0.2 ps [43], the solvent reorganizes immediately to the new electronic configuration upon electronic excitation. Consequently, all of the observed spectra for 4-AP in CD_3_CN exhibited a fully optimized solvent configuration in the electronic state; thus, all spectra recorded in acetonitrile were denoted as S_n_.

Time-dependent amplitude changes in the basis spectra, obtained from global fitting of the TRIR spectra, were analyzed in the same manner as for 4-AP in methanol using Figure 6b. Since CD_3_CN does not form hydrogen bonds with 4-AP, and such bonding is required to enable the ESIPT of 4-AP, the enol form is not produced. As shown in Figure 9b, the time-dependent fractional population changes in all species involved in the photoexcitation of 4-AP in CD_3_CN at 300 nm are well reproduced by Figure 6b shown in Figure 6b.

According to Figure 6b, all 4-APs in the 300 nm excited S_2_ state decay to the S_1_ state via internal conversion with a time constant of 9 ± 2 ps, while the 4-AP in the S_1_ state decays to the S_0_ state with a time constant of 10 ± 2 ns, indicating that the excited state life time of the S_1_ state of 4-AP in CD_3_CN is 10 ± 2 ns. This S_1_ lifetime is consistent with the reported value of 14 ns measured using the fluorescence decay time of 4-AP in CH_3_CN excited at 375 nm [22]. When excited to the S_2_ state, 4-AP undergoes the same dynamics in both protic and aprotic solvents, namely simple relaxation to the ground state via the S_1_ state. These results, therefore, confirm that no ESIPT proceeds even for excitation to the S_2_ state.

## 3. Experimental Section

### 3.1. Sample Preparation

4-AP, CH_3_OH, CD_3_OD, CH_3_CN, and CD_3_CN were purchased from Sigma-Aldrich (St. Louis, MO, USA) and were used without further purification. Solutions of 4-AP (10 mM) were prepared in methanol and acetonitrile under N_2_ gas to avoid moisture contamination. The equilibrated 10 mM 4-AP solutions were loaded into a gas-tight sample cell (100 μm pathlength) with two 2 mm-thick CaF_2_ windows. The concentration of 4-AP and pathlength of the cell were tuned to obtain the maximum transient absorption signal. UV-Vis and FT-IR spectra were recorded throughout the experiments to ensure sample integrity. All TRIR experiments were performed at 293 ± 1 K.

### 3.2. Time-Resolved Mid-IR Spectroscopy

The mid-IR transient absorption spectra were measured using a femtosecond temporal resolution according to the pump-probe technique. Details of the TRIR spectrometer employed herein are described elsewhere [30,31,32,33]. A commercial Ti:sapphire regenerative amplifier (Spitfire Ace, Spectra Physics, Milpitas, CA, USA) was used, which produces 120 fs laser pulses at 800 nm with a repetition rate of 2 kHz. The amplified 800 nm pulse was split and used to pump two homemade optical parametric amplifiers (OPAs). A tunable mid-IR pulse (100 fs, 160 cm^−1^, 1 μJ) was generated by difference frequency mixing of the near-IR signal and the idler pulses of one OPA. A tunable UV (370–300 nm, 160 fs, 3 μJ) pulse was generated by frequency doubling the visible pulse, which was itself produced by frequency doubling the near-IR signal pulse of the other OPA. A translational stage was used to control the optical delay between the mid-IR probe and the UV pump pulses up to 1 ns. Since an optical delay beyond 1 ns is impractical, the pump pulse was replaced with the output from a commercial nanosecond tunable laser (pulse width = 2.5 ns; NT240, EKSPLA, Vilnius, Lithuania), which was synchronized with the femtosecond mid-IR probe pulse using an electronic digital delay generator (DG535, Stanford Research Systems, Sunnyvale, CA, USA). The sample was excited with a UV pulse of 1–3 μJ and probed with a small portion of the femtosecond mid-IR pulses (~10 nJ). To acquire an isotropic absorption spectrum, the polarization of the pump pulse was set at the magic angle (54.7°) relative to that of the probe pulse. The mid-IR pulse was centered at either 1750, 1650, 1580, or 1520 cm^−1^ to probe a broader spectral region than the spectral width (160 cm^−1^) of the pulse. The broadband mid-IR pulses passed through the sample and were then dispersed in a 320 mm monochromator equipped with a 50 lines/mm grating and a single HgCdTe array detector with 1 × 64 pixels, resulting in a spectral resolution of ~1.5 cm^−1^ per pixel. The signals obtained from 64 pixels for each probe pulse were amplified and digitized using 16-bit A/D converters to determine the transmitted light intensity at the corresponding wavelength. A synchronous optical chopper operating at 1 kHz was used to block alternate pump pulses at 2 kHz, allowing measurement of the pump-induced absorbance change at each wavelength based on the adjacent transmitted intensities, with and without excitation. In addition, in contrast to blocking the other pulses operating at 2 kHz, the nanosecond pump laser was operated at 1 kHz. The transient absorption of the Si wafer was used to determine the instrument response function: 0.2 ps to 1 ns with the femtosecond pump pulse, and 2.5 ns over 1 ns with the nanosecond pump pulse.

### 3.3. Computational Details

All quantum calculations were performed using the DFT approach with the ωB97XD functional and the pcseg-1 basis set in the Gaussian 16 software. The solvent effect was incorporated using a polarizable continuum model. The vertical excitation energies, oscillator strengths, molecular geometry optimization, and vibrational frequency of the excited states (S_n_, n ≥ 1) for 4-AP were performed within the TD-DFT framework at the ωB97XD/pcseg-1 level. The TD-DFT calculations showed that pump wavelengths of 300 and 350 nm could lead to electronic transitions in 4-AP, generating the electronically excited S_2_ and S_1_ states, respectively. The vertical excitation energies, molecular geometry optimization, and vibrational frequency of the S_1_ state of Enol 4-AP were also computed using the same approach. The scaling factor (0.923–0.975) was adjusted to match the calculated vibrational frequencies of 4-AP with the positions of the bands, thereby forming the basis functions used to fit the TRIR spectra of 4-AP.

## 4. Conclusions

In this study, the dynamics of excited 4-aminophthalimide (4-AP) in methanol and acetonitrile were investigated after excitation at 300 and 350 nm using femtosecond time-resolved infrared (TRIR) spectroscopy. Upon excitation to the S_2_ state, 4-AP undergoes internal conversion to the S_1_ state with time constants of 9–13 ps and subsequently relaxes from the S_1_ state to the ground state with time constants of 6–14 ns. Upon excitation to the S_1_ state, 4-AP relaxes to the ground state on the nanosecond scale. The energy of the enol form of 4-AP (Enol 4-AP) in the S_1_ state was calculated to be ~10 kcal/mol higher than that of the keto form in the S_1_ state, indicating that the excited-state intramolecular proton transfer (ESIPT) of 4-AP in the S_1_ state is an endergonic process. This accounts for the fact that no ESIPT was observed upon the excitation of a protic solution of 4-AP to the S_1_ state. Although the transition from the 4-AP S_2_ state to the Enol 4-AP S_1_ state was exergonic, no ESIPT was observed since rapid internal conversion from the keto S_2_ to S_1_ states dominated the relaxation process. Overall, using structure-sensitive TRIR spectroscopy, detailed dynamics were obtained for photoexcited 4-AP in solution, revealing the molecular mechanism for the negligible ESIPT of 4-AP, despite the fact that 4-AP forms an optimal six-membered structural configuration that is conducive to ESIPT.

## Figures and Tables

**Figure 7:**
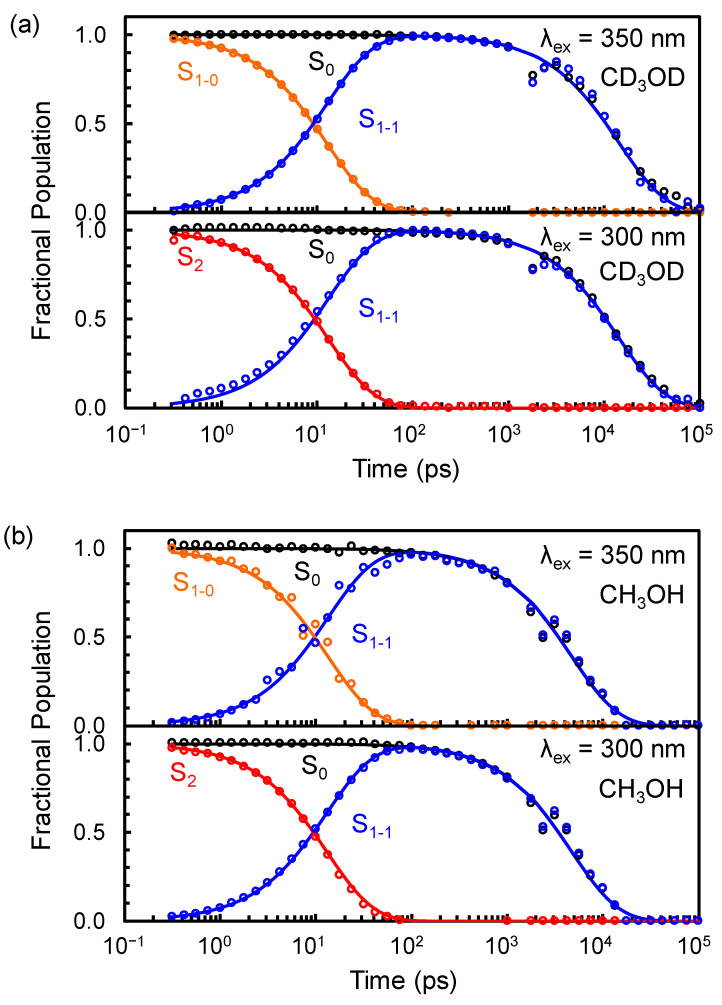
Time-dependent fractional population changes in 4-AP in various electronic states in (**a**) CD_3_OD and (**b**) CH_3_OH after excitation at 350 and 300 nm. The fractional population changes are color-coded: orange, blue, and red circles and lines represent the fractional population of 4-AP in methanol for the S_1-0_, S_1-1_, and S_2_ states, respectively. Black circles and lines represent the fractional population change in the depleted 4-AP after excitation. Data for the fractional population changes (open circles), obtained from the time-dependent amplitude changes in the basis spectra and the fitted integrated extinction coefficients of the corresponding basis spectra, were well described by Figure 6b with the fitted time constants shown in Figure 6b (solid lines).

## Data Availability

Data are contained within the article.
